# Early-Onset Progressive Retinal Atrophy Associated with an *IQCB1* Variant in African Black-Footed Cats (*Felis nigripes*)

**DOI:** 10.1038/srep43918

**Published:** 2017-03-21

**Authors:** Annie Oh, Jacqueline W. Pearce, Barbara Gandolfi, Erica K. Creighton, William K. Suedmeyer, Michael Selig, Ann P. Bosiack, Leilani J. Castaner, Rebecca E. H. Whiting, Ellen B. Belknap, Leslie A. Lyons, 99 Lives Consortium, Danielle Aderdein, Paulo C. Alves, Gregory S. Barsh, Holly C. Beale, Adam R. Boyko, Marta G. Castelhano, Patricia Chan, N. Matthew Ellinwood, Dorian J. Garrick, Christopher R. Helps, Christopher B. Kaelin, Tosso Leeb, Hannes Lohi, Maria Longeri, Richard Malik, Michael J. Montague, John S. Munday, William J. Murphy, Niels C. Pedersen, Max F. Rothschild, William F. Swanson, Karen A. Terio, Rory J. Todhunter, Wesley C. Warren

**Affiliations:** 1 Department of Veterinary Medicine and Surgery, College of Veterinary Medicine, University of Missouri, Columbia, Missouri, USA; 2 Kansas City Zoo, Kansas City, Missouri, USA; 3 Cleveland Metroparks Zoo, Cleveland, Ohio, USA; 4 Animal Eye Care of Richmond LLC, Midlothian, Virginia, USA; 5 Department of Ophthalmology, University of Missouri School of Medicine, One Hospital Drive, Columbia, MO, 65212, USA; 6 Metropolitan Veterinary Referral Hospital, Akron, Ohio, USA; 7 Institute of Veterinary, Animal and Biomedical Sciences, Massey University, Palmerston North 4474, New Zealand; 8 CIBIO/InBIO, Centro de Investigação em Biodiversidade e Recursos Genéticos, Universidade do Porto, Campus Agrário de Vairão, 4485–661, Vairão, Portugal; 9 Departamento de Biologia, Faculdade de Ciências da Universidade do Porto (FCUP), 4169–007 Porto, Portugal; 10 Wildlife Biology Program, University of Montana, Missoula, Montana, 59812, USA; 11 HudsonAlpha Institute for Biotechnology, Huntsville, Alabama 35806, USA; 12 Department of Genetics, Stanford University, Stanford, California, 94305, USA; 13 Maverix Biomics, Inc., San Mateo, California, 94402, 695, USA; 14 Department of Biomedical Sciences, College of Veterinary Medicine, Cornell University, Ithaca, New York, 14853, USA; 15 Department of Clinical Sciences, College of Veterinary Medicine, Cornell University, 700 Ithaca, New York, 14853, USA; 16 Department of Animal Science, College of Agriculture and Life Sciences, Iowa State University, Ames, Iowa, 50011, USA; 17 Department of Pathobiology, Institute of Veterinary, Animal and Biomedical Sciences, Massey University, Palmerston North 4474, New Zealand; 18 Langford Veterinary Services, University of Bristol, Langford, Bristol, BS40 5DU, UK; 19 Vetsuisse Faculty, Institute of Genetics, University of Bern, Bern, Switzerland; 20 Swiss Competence Center of Animal Breeding and Genetics, University of Bern, 3001, Bern University of Applied Sciences HAFL & Agroscope, 3001, Bern, Switzerland; 21 Department of Veterinary Biosciences and Research Programs Unit, Molecular Neurology, University of Helsinki and Folkhälsan Research Center, Helsinki 00014, Finland; 22 Dipartimento di Scienze Veterinarie e Sanità Pubblica, University of Milan, 20122 Milan, Italy; 23 Centre for Veterinary Education, University of Sydney, Sydney, NSW 2006, Australia; 24 The Genome Institute, Washington University School of Medicine, St. Louis, Missouri, 63108, USA; 25 Department of Veterinary Integrative Biosciences, College of Veterinary Medicine, Texas A&M University, College Station, Texas, 77845, USA; 26 Department of Medicine and Epidemiology, School of Veterinary Medicine, University of California at Davis, Davis, California, 95616, USA; 27 Center for Conservation and Research of Endangered Wildlife (CREW), Cincinnati Zoo & Botanical Garden, Cincinnati, Ohio, 45220, USA; 28 Zoological Pathology Program, University of Illinois, Maywood, IL 60153, USA

## Abstract

African black-footed cats (*Felis nigripes*) are endangered wild felids. One male and full-sibling female African black-footed cat developed vision deficits and mydriasis as early as 3 months of age. The diagnosis of early-onset progressive retinal atrophy (PRA) was supported by reduced direct and consensual pupillary light reflexes, phenotypic presence of retinal degeneration, and a non-recordable electroretinogram with negligible amplitudes in both eyes. Whole genome sequencing, conducted on two unaffected parents and one affected offspring was compared to a variant database from 51 domestic cats and a Pallas cat, revealed 50 candidate variants that segregated concordantly with the PRA phenotype. Testing in additional affected cats confirmed that cats homozygous for a 2 base pair (bp) deletion within *IQ calmodulin-binding motif-containing protein-1 (IQCB1*), the gene that encodes for nephrocystin-5 (NPHP5), had vision loss. The variant segregated concordantly in other related individuals within the pedigree supporting the identification of a recessively inherited early-onset feline PRA. Analysis of the black-footed cat studbook suggests additional captive cats are at risk. Genetic testing for *IQCB1* and avoidance of matings between carriers should be added to the species survival plan for captive management.

Whole genome (WGS) or whole exome sequencing (WES) can identify *de novo* or private variants causing a disease, which allows clinicians to better predict individualized effective treatments^1^,^2^. Even if clinically relevant genetic variation remains a challenge to define, WGS/WES of small groups of related individuals, such as trios, parent-offspring, affected duos, have proven powerful to identify *de novo* or rare variants for orphan diseases in many species^3^,^4^,^5^. However, WGS/WES approaches are more limited if a disease is caused by a combination of common and or rare variants and, especially, if the variants are in non-coding, regulatory regions of a gene. Thus, only ~30–40% of patients may be diagnosed by WGS/WES^6^,^7^. The power of WGS/WES causal variant identification improves when the database of genome variants is from a variety of individuals, ethnic groups (breeds) and racial populations and when precise clinical data is available for each individual^8^,^9^,^10^.

The state-of-the art health care provided by WGS/WES is now available for domestic cats via the 99 Lives Cat Genome Sequencing Initiative (http://felinegenetics.missouri.edu/99lives)^11^,^12^. However, the application of WGS to endangered felid species for health care management has not been attempted previously. Captive felid populations have limited numbers of founders and health problems can easily arise as a result of deleterious mutations carried by one or more of the founders. Although widely available for domestic animals and humans, genetic tests for inherited disease are not readily available for captive felids. The identification of the rare color variants for white lions^13^ and white tigers^14^ could be used to outbreed these cats and reduce recognized inbreeding depression, however, genetic testing for these variants have not been implemented into species survival plans. Accessibility of genetic tests for diseases recognized and observed in zoo populations may prove to be critical for the long-term survival of species that are threatened with extinction and are managed in captive breeding programs.

The black-footed cat (*Felis nigripes; Fnig;* Burchell, 1824) (Fig. 1a) is the smallest wild cat found in Africa, endemic to the arid steppe and savannah habitats of the southern African subregion^15^. Phylogenetic studies indicate that the black-footed cat is part of the domestic cat lineage along with several other wild felids, including the European wildcat (*Felis silvestris silvestris*), African wildcat (*Felis silvestris lybica*), Chinese desert cat (*Felis bieti*), Sand cat (*Felis margarita*), and Jungle cat (*Felis chaus*) (Fig. 1b)^16^,^17^,^18^. Unfortunately, the size of both the wild and captive populations (~50 individuals in zoos worldwide) are diminishing. The species is listed under Appendix I–threatened with extinction–by the Convention on the International Trade of Endangered Species (CITES)^19^, as “vulnerable” by the International Union for Conservation of Nature (IUCN) since 2002^20^, and as “Endangered” by the United States Fish and Wildlife Service^21^. Black-footed cats typically have shorter life spans in captivity than in the wild. The majority of captive adult black-footed cat deaths occur between 2 to 5 years, whereas free-ranging cats have been estimated to live approximately 5 to 6 years^22^. Recent reports have documented that renal amyloidosis plays an important role in the decreased survival of captive black-footed cats^23^. Information concerning other disease processes or conditions that occur in wild or captive black-footed cats is limited. Due to their endangered status and their relatively small captive breeding population, the presence of genetic defects, such as heritable blindness, could be detrimental for the long-term sustainability of captive black-footed cat populations.

Progressive retinal atrophy (PRA) refers to inherited retinal dystrophic or degenerative disorders present in animals. The age of onset and rate of progression of initial photoreceptor death varies with each species and each type of PRA. Initially, the disease usually affects the photoreceptor layer (specifically the rods) and proceeds to affect all retinal layers over time leading to diffuse retinal degeneration and ultimately blindness^24^. Four different types of PRA have been described in the domestic cat (*Felis catus*)^25^,^26^,^27^,^28^. In Abyssinians, an autosomal dominant early-onset cone-rod dystrophy (*rdy*) manifests from a mutation in *CRX*^27^,^29^,^30^,^31^,^32^,^33^, while autosomal recessive late-onset rod-cone degeneration (*rdAc*) results from a mutation in *CEP290*^26^,^34^,^35^,^36^,^37^,^38^. In Persians, an autosomal recessive early-onset rod-cone dystrophy has been described and a causal variant in *AIPL1* detected^28^,^39^,^40^. In Bengal cats, an autosomal recessive PRA has been characterized^25^. Due to their similarity to human globe size, relatively accelerated lifespan and different represented modes of inheritance for PRA, domestic cats represent a useful animal model for retinal diseases in humans such as retinitis pigmentosa and Leber’s congenital amaurosis^41^.

The present study describes an early-onset generalized PRA diagnosed in two full-sibling African black-footed cats (*Felis nigripes*). Clinical abnormalities in affected cats were present as early as 3 months of age. This is the first report of ocular disease in this species. A novel cross-species approach was used for the genetic analyses to rapidly identify a highly associated causal variant. Variants within the 99 Lives cat genome sequence database, which contains whole genome sequences of 51 domestic cats at the time of the study, were compared to the variants identified in the black-footed cats. Variants were prioritized as specific to the Black-footed cat and segregating within the trio with the PRA phenotype. A DNA-based test for management of the disease within the captive breeding program can now be implemented.

## Results

### Ophthalmic examination and diagnostic results

**Figure 2:**
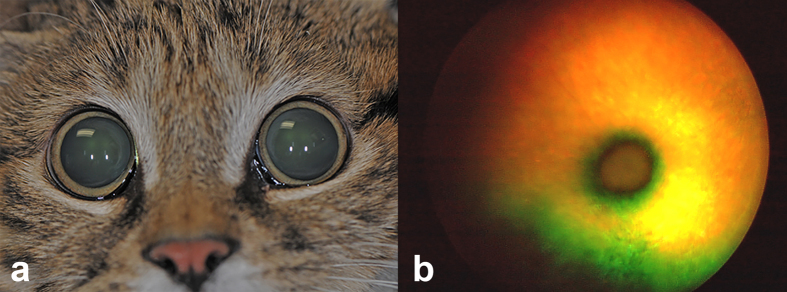
Extraocular view of the affected male proband black-footed cat (*Felis nigripes*). (**a**) The image demonstrates marked mydriasis in ambient light. (**b**) Color fundus photograph from the right eye of the affected male proband demonstrates diffuse vascular attenuation and tapetal hyperreflectivity consistent with progressive retinal atrophy in cats.

No significant abnormal findings were observed on physical assessment of the male proband cat. However, both pupils were maximally dilated in well-lit conditions, circular in shape, and unresponsive to light (Fig. 2a). Menace responses and dazzle reflexes were absent, and significantly reduced direct and indirect PLRs were observed bilaterally. The cat did not show appropriate tracking responses. Palpebral reflexes, corneal sensitivity and oculocephalic reflexes were normal. On slit-lamp biomicroscopy, the eyelids, conjunctiva, and anterior chamber were normal. Other abnormalities noted included faint linear superficial dendritic corneal facets and a relucency of the embryonic lens nucleus in the left eye. Indirect ophthalmoscopy identified generalized tapetal hyperreflectivity and severe vascular attenuation bilaterally (Fig. 2b) consistent with end-stage PRA. Schirmer I tear test values (mean ± standard deviation) were 5 mm/min in both eyes, which are decreased compared to the normal reference range for domestic felids (14.3 ± 5.73 mm/min)^42^,^43^. Bilaterally decreased Schimer I tear test values was most likely secondary to sedation^44^,^45^. Intraocular pressure (IOP) data (mean ± standard deviation) collected with rebound tonometry revealed 17 mmHg bilaterally, which was within the normal reference interval for domestic felids (22.6 ± 4.0 mmHg)^46^. The cornea was fluorescein negative in both eyes. Physical, ophthalmic, and neuro-ophthalmic examination of the female sibling was similar to the male proband. Schirmer I tear test values were decreased in the right (3 mm/min) and left (5 mm/min) eye, presumably due to the influence of general anesthesia. The IOP measured 21 mmHg and 24 mmHg in the right and left eye, respectively.

In both the male proband and its female full-sibling, the electroretinogram (ERG) was non-recordable with a “flat line” in both eyes, consistent with end-stage PRA. Specific photoreceptor function could not be evaluated due to the flat (non-recordable) waveform in both cats. Complete blood hematology, chemistry, and urinalysis were unremarkable, and plasma taurine levels were within normal reference intervals in both cats. Indirect blood pressure measurements obtained under general anesthesia in both cats were found to be within normal reference ranges for domestic felids^47^,^48^,^49^.

### *CEP290, CRX*, and *AIPL1* genetic testing

Genetic testing of the black-footed female proband and its two unaffected parents was performed at the commercial service at the Veterinary Genetics Laboratory (VGL) at the University of California–Davis. The cats were confirmed to be wild type for the variants known to cause PRA in Abyssinian type domestic cats, specifically the variants within *CRX*^27^ and *CEP290*^26^, and in Persian type domestic cats within the *AIPL1* gene^12^.

### Variant analysis

**Figure 3:**
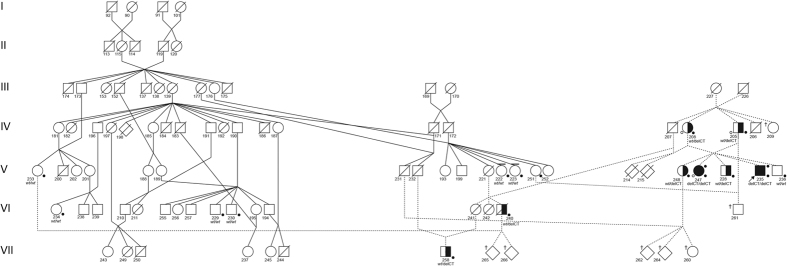
Pedigree of African black-footed cats with progressive retinal atrophy. The pedigree represents all living cats (<50) in the North American studbook, cats with available DNA samples, and related individuals. Circles represent females, squares represent males and diamond represents cats of unspecified gender. Open symbols indicate phenotypically *wild type* (normal) cats. Solid symbols indicate PRA-affected cats. Half-filled symbols indicate obligate carrier cats. Diagonal lines across symbols represent deceased cats. A solid dot above right of the symbol indicates cats that underwent ophthalmic (Schirmer tear test I, rebound tonometry, fluorescein stain, slip-lamp biomicroscopy, funduscopy, and ERG) and neuro-ophthalmic (direct and consensual pupillary light reflexes, dazzle reflex, menace response, and measurement of horizontal pupil diameter) examination. Open dot below left of symbol indicates the trio of cats that were whole genome sequenced. The arrow indicates the male proband. A solid dot below right of symbol indicates cats with available DNA (blood or hair) specimens (n = 16) and were genotyped. The genotype is unknown for cats not sampled. Genotype are presented below the cat symbols: *IQCB1* deleted 2 base pairs cytosine and thymine (delCT), *wild type (wt*), *IQCB1-*mutant (delCT/delCT), *IQCB1-*carrier (*wt/*delCT), *wild type (wt/wt*). Dotted lines represent the lineage of black-footed cats that segregate for vision impairment and the cross over that occurred to another line of breeding of cats. Cross symbol (†) indicates cats with increased risk for affected or carrier status. Line above the symbol indicates black-footed cats that are carriers for variants for the genes *CDH23-* and *DTHD1_44* based on mass spectometrocopy genotyping. Since these cats never interbred with the lineage that segregates for the vision impairment, *CDH23* and *DTHD1_144* can be excluded as candidate genes for PRA in African black-footed cats. Seven cats in the studbook are at risk for perpetuating PRA in the black-footed cat captive breeding program.

WGS was conducted on a PRA affected female offspring (247) and its obligate carrier sire (205) and dam (208) (Table 1, Fig. 3). Average WGS coverage for each individual was 30X. The available database used for variant filtering included variants from 52 individuals, 51 domestic cats and one opportunistic Pallas cat, each with an average 30X genome coverage. Over 22 million variants were identified when the WGS of the trio of cats was aligned to the available annotated cat genome reference (Felis_catus 6.2 genome assembly)^50^. After excluding variants in common between the three black-footed cats and one Pallas cat (*Otocolobus manul*), over 13 million were identified. Thus, the one Pallas cat eliminated 41% of identified variants that were common between the two wild felid species and found in domestic cats. Over 234,000 variants segregated concordantly when considering the phenotype within the trio and uniqueness to black-footed cats. Only fifty variants had a predicted high impact on a protein product, including, eight predicted stop gains, 20 predicted splice donor-acceptors and predicted 22 frameshift variants. The prioritization of the variants by filtering and impact is presented in Table 2.

A list of over 240 genes associated with blindness is available on the RetNet database (www.RetNet.org). Of 50 genes with high impact variants, only three genes (*RASGRF1, IQCB1* and *CCDC114*) had been previously associated with blindness (Supplementary Table 1). The *RASGFR1* and *CCDC114* variants were associated with several complex clinical features including retinal atrophy, whereas the phenotype associated with the *IQCB1* variant was always PRA with extrarenal manifestations in 10–15% of cases. The high impact variant detected within the coding region of *IQCB1* is a 2 base pair (bp) deletion in exon 13 (c.1282delCT) and was considered the highest priority. The identified 2 bp variant was homozygous for the deletion in the affected cat and heterozygous in both phenotypically normal parental obligate carriers (Table 3). The polymorphism is predicted to cause a protein truncation at position 428 of the *IQCB1* amino acid chain (p.L428*), truncating the last 170 amino acids at the C-terminal of nephrocystin-5 (NPHP5). A pedigree for all living cats (<50) in the North American black-footed cat studbook, cats with available DNA samples, and related individuals is shown (Fig. 3). The identified c.1282delCT was screened via direct sequencing in 16 available samples; blind individuals (n = 2) tested homozygous for the mutated allele while sighted individuals (n = 13) were heterozygous (n = 5) or homozygous (n = 8) wild type. One sample failed to amplify.

In addition, seven moderate impact missense variants were identified in RetNet genes that were unique to black-footed cats and segregated concordantly with the disease phenotypes of the WGS trio (Table 2). Those seven genes were not on the same chromosome as the putative causal *IQCB1.* The polymorphisms within *CHD23, CDH3, CNGB1, DTHD1 (2 variants), RBP3* and *USH1C* were investigated in the additional black-footed cats using mass spectrometry (Table 3). Five of seven variants (*CHD3, CNGB1, DTHD1_942, RBP3,* and *USH1C*) were excluded because the cats’ genotype was discordant with PRA phenotype, assuming a recessive mode of inheritance model as suggested by pedigree analysis. Specifically, these variants were either not homozygous in the second PRA-affected cat or homozygous in other sighted black-footed cats. The remaining two missense variants (*CDH23* and *DTHD1_144*) could not be excluded based on the genotype and phenotype discordance but were heterozygous each in six sighted cats from the pedigree. These variants were considered less likely associated with the phenotype because the variant alleles in *CDH23* and *DTHD1_144* were detected in unrelated black-footed cats that have not been interbred with the lineage that segregates for vision impairment (Fig. 3). For example, a visually sighted cat (223) is a *DTHD1_144-*carrier and this cat has no relationship to the affected lineage. WGS also confirmed that the cats of the trio are wild type for the known mutations within *AIPL1, CRX* and *CEP290*, confirming the commercial service genetic testing.

## Discussion

The genetics of the patient is a critical aspect of an individual’s health care for some diseases. The reduced cost of WGS and WES has made feasible the examination of entire genomes for individualized healthcare. Animals now have the same benefit since lower sequencing costs have allowed the development of DNA variant databases from normal control animals for comparison to individuals with diseases. The study of rare, simply inherited recessive diseases particularly benefit from the WGS approach. Although a large database of normal DNA variants within the genome of black-footed cats is not available, because these wild felids are phylogenetically closely related to domestic cats, the domestic cat genome variant database was used for comparison of polymorphisms to exclude variants in common between the species. Herein is the first cross-species comparison of WGS data to support the captive management of an endangered species.

Vision loss is a significant concern in the vulnerable African black-footed cat as poor eyesight could result in a poor quality of life and failure to thrive for individuals, impacting healthy captive breeding practices for the species as a whole. Clinical examination of the affected male proband and female sibling cat revealed findings consistent with PRA. The ERG confirmed that both cats retained minimal or no retinal function, consistent with end-stage PRA. Clinical abnormalities in affected cats were present as early as 3 months of age and rapidly progressed to end-stage PRA, supporting an early-onset retinal disorder affecting both rod and cone photoreceptors. The male proband and female cat are full-siblings, but from different litters. Pedigree analysis supported an autosomal recessive mode of inheritance since visually normal parents produced both affected (n = 2) and unaffected kittens (n = 3), the parents of affected kittens had consanguinity and the affected kittens were either male (n = 3) and female (n = 2) (Fig. 3).

Results of ophthalmic examination and testing, pedigree analysis, and lack of evidence for nutritional, toxic, and hypertensive etiology for the retinal degeneration in the affected African black-footed cats strongly suggested a genetic basis for early-onset PRA. The male proband and female sibling cat were identified at 7 and 3 months of age, respectively, however initial signs of compromised photoreceptor integrity and function may have occurred at an earlier time point. The phenotype and electrodiagnostic findings in PRA-affected black-footed cats are chronologically most consistent with early-onset PRAs in Persians and Bengals, and inconsistent with autosomal dominant early-onset PRA of *CRX (rdy*) and autosomal recessive late-onset PRA of *CEP290 (rdAc*)-mutant Abyssinians. Although the presence of a domestic cat variant for vision loss would be highly unlikely in these black-footed cats, genetic testing for cat PRAs is rapid and low cost. Genotyping excluded the Persian autosomal recessive early-onset *AIPL1* variant as well as Abyssinian *rdAc* and *rdy* PRA variants. The WGS would allow more exhaustive consideration of these same genes for a species-specific variant.

To identify the causal gene, WGS of a trio of cats, comprising two normal obligate carrier parents, and one affected offspring, was conducted. This study of the single black-footed cat was assisted by comparison variants from the closely related domestic cat database, which included variants from 51 domestic cats and one Pallas cat (*Otocolobus manul*) at the time of the study. The Pallas cat was an opportunistic inclusion in the 99 Lives dataset, included as part of a separate study focusing on polycystic kidney disease. The Pallas cat is the next nearest feline lineage and did support the filtering of shared wild felid variants and shared variants with domestics that would not be causal. Forty-one percent of variants were eliminated by the cross-species comparison. One of the fifty high impact variants resided in a gene known to cause retinal degeneration (*IQCB1*) and listed on RetNet. A 2 bp deletion within the coding region of the black-footed cats *IQCB1* was detected and segregated concordantly with the disease phenotype (Fig. 3). The mutation is predicted to cause a protein truncation at position 428 of the amino acid chain (p.L428*), deleting an IQ calmodulin-binding protein domain and the complete protein C-terminal^51^. Seven moderate impact missense variants were also detected in RetNet genes that were unique to the three sequenced black-footed cats and segregated with the disease. Mismatch genotype and phenotype in addition to the probability of an unrelated cat being affected excluded five of the missense variants as potential candidate genes. Additionally, the remaining two variants were most likely not associated with the retinal degeneration since they were present in individuals unrelated to the affected cats. Although more likely not associated, the variants within *CDH23* and *DTHD1_144* will be tested along with the *IQCB1* 2 bp deletion in all samples that will be received in the future, until when excluded or confirmed by the phenotype.

Variants in *IQCB1 (IQ calmodulin-binding motif-containing protein-1*) are known to historically cause Senior-Loken syndrome (SLSN) in humans, a rare autosomal recessive disease characterized by both nephronophthisis (NPHP) and retinal degeneration^51^,^52^. All affected human patients with SLSN exhibit Leber congenital amauorsis (LCA). Leber congenital amauorsis is generally inherited in an autosomal recessive manner and associated with over 21 gene variants in animals and humans related to retinal function, including *RPE65, CRX, CEP290, AIPL1,* and *IQCB1*^53^. The *IQCB1* gene encodes nephrocystin-5 (NPHP5), one of thirteen ciliary proteins collectively known as nephronophthisis (NPHP) proteins^54^. The NPHP5 protein is present in the photoreceptor connecting cilium, a zone that connects the photoreceptor cell body to the outer segments, where *IQCB1* Interacts with *CEP290*^51^,^55^. The ciliary protein contains two calmodulin binding sites and co-localizes with nephrocystin-1, nephrocystin-4^51^. The NPHP5 protein also interacts with nephrocystin-6^55^ and retinal ciliopathy gene retinitis pigmentosa GTPase regulator (RPGR)^51^. Premature terminating mutations in *NPHP5* lead to a truncated protein, which disrupts peptide transportation, compromising the structural and functional integrity of the photoreceptor, leading to cone and rod death and subsequent retinal degeneration^51^,^55^. Variants in NPHP proteins also cause a broad-spectrum of autosomal recessive kidney diseases that are the most frequent genetic cause of end-stage kidney disease in children and young adults^56^. Extra-renal manifestations occur in 10–15% of cases and retinal degeneration are the most common abnormality. Thus, kidney function of PRA-affected black-footed cats should be monitored.

*IQCB1* variants have not been recognized in the domestic cat but an insertional mutation in *NPHP5 (IQCB1)* has been recently documented in American Pit Bull Terrier dogs with early-onset recessively inherited cone-rod dystrophy (*crd2*)^57^,^58^. In mutant dogs, the resulting ciliopathy causes early loss of rod photoreceptors and relative retention of central retinal cone photoreceptors that lack function^58^. In the affected dogs, non-ophthalmic problems were neither observed nor reported. Similarly, a variant in *NPHP4* is associated with recessive cone-rod dystrophy in Standard Wire-haired Dachshunds. No kidney involvement has been reported in affected dachshunds^59^.

The pedigree reveals three unique lineages in the African black-footed cat captive population (Fig. 3). The *IQCB1*-mutant gene was isolated within one lineage, but has crossed to another lineage with a recent breeding of cats. Ideally, the *IQCB1* variant should be eliminated within the breeding population of captive black-footed cats. This would require all living cats (<50) to be genotyped. Specifically cats with an increased risk for affected or carrier status based on the pedigree analysis and known genotypes (Fig. 3; 209, 260, 261, 262, 264, 265, and 266). *IQCB1-*carriers (or *CDH23* and *DTHD1* carriers) would need to be mated with *wild type* cats. If significant population changes occurred in African black-footed cats that necessitated breeding of *IQCB1*-mutant cats from a conservation standpoint, they should be bred to *wild type* individuals to maintain maximum genetic diversity within this species. Additionally, the black-footed cats related to the founder cats of the PRA lineage could be genotyped to prevent further influx of the variant into captive breeding programs worldwide.

This is the first report of early-onset recessively inherited PRA in the African black-footed cat. Genetic analysis revealed the etiology to likely be a frameshift mutation in the *IQCB1* gene, or a missense mutation within *CDH23* or *DTHD1. IQCB1* represents the most likely variant associated with the phenotype, and the identified polymorphism is predicted to code for a truncated NPHP5 protein in the connecting cilium which is present in both rod and cone photoreceptors. Genetic testing all the candidate variants in more affected and sighted individuals will help confirm the causal variant associated with the PRA. The identification of the putative causal gene provides a DNA-based test for appropriate captive breeding practices and conservation of this vulnerable wild felid. Moreover, the WGS variant database comprised of 51 domestic cats and one Pallas cat at the time of the study has proven useful for cross-species comparisons for species that have recent common ancestors.

## Methods

### Ethical statement

All procedures used to evaluate the animals’ health were performed during the animals’ routine health screening at each participating zoo. Direct ophthalmoscopy and phlebotomy via jugular venipuncture were performed as part of the complete physical exam on each individual. The main purpose of the blood specimen collection was to evaluate overall systemic health, and surplus EDTA anti-coagulated whole blood was submitted for genetic analyses. This study was completed in strict accordance with the recommendations from the Kansas City Zoo and Cleveland Metroparks Zoo Scientific Committees. Experimental protocols and applications for use of biomaterials were approved by the Kansas City, Cleveland Metroparks and Omaha’s Henry Doorly Zoo Research Review Committees. Examinations, blood or hair specimen collection, and advanced diagnostics were performed under sedation or general anesthesia. Samples were received for the genetic analyses under the University of Missouri Institutional Animal Care and Use Committee protocol 8313. Blood or hair samples submitted by other participating zoological parks were collected opportunistically during the cat’s animal health exam and were submitted for diagnostic purposes.

### Animals and Clinical descriptions

The 7 month-old neutered male proband African black-footed cat from the Kansas City Zoo presented to the University of Missouri, College of Veterinary Medicine (MU CVM), Columbia, MO for suspected blindness, dilated pupils, absent dazzle reflex in both eyes, and inappropriate tracking of moving objects during quarantine examination. Mild horizontal nystagmus was also present in the right eye. Before presentation, the cat had been screened and treated for parasites, vaccinated for feline viral rhinotracheitis, calicivirus, and panleukopenia (FVRCP) (Boehringer Ingelheim, St. Joseph, MO) in addition to Purevax Ferret Distemper (Merial Inc., Athens, GA) and was considered otherwise healthy. The cat was not receiving any medications and had never been administered fluoroquinolones. Daily diet provided once a day included Evo turkey and chicken formula (15 grams), Nebraska Canine Premium (40 grams) and a small mouse.

From a different litter, the 3 month-old intact female sibling African black-footed cat from the Cleveland Metroparks Zoo presented to the Metropolitan Veterinary Hospital, Akron, OH for fixed and dilated pupils and vision deficits bilaterally. The female sibling had a history of poor weight gain that resolved with treatment and increased caloric intake. On presentation, the cat had received its first vaccination for feline viral rhinotracheitis, calicivirus, panleukopenia, and chlamydia (FVRCP-C) (Boehringer Ingelheim, St. Joseph, MO), was not on any medications, and had never been administered fluroquinolone antibiotics. Daily diet included Nebraska Brand Premium Feline Diet (75 grams) and a mouse (25 grams). Both cats were housed indoors in an approximately 10 × 10 foot enclosure under a 12 hour light - dark cycle.

### Ophthalmic examination

Both affected African black-footed cats underwent complete physical, ophthalmic and neuro-ophthalmic examinations. Assessment of the male proband cat was completed under sedation and physical restraint by a board-certified veterinary ophthalmologist (JWP) and board-certified zoo veterinarian (WKS) at the MU CVM. Sedation was achieved with an intramuscular injection of ketamine (Pfizer, New York, NY, USA; 7 mg) and dexmedetomidine (Pfizer, New York, NY, USA; 0.55 mg), and 100% oxygen was provided via mask. A 24-gauge catheter was placed in the saphenous vein and Lactate Ringer’s Solution (LRS) was administered at a rate of 10 ml/kg/hour. The animals’ vital signs (heart rate, respiratory rate, and temperature) were evaluated every 5 minutes. Following the procedure, the sedation was reversed with an intramuscular injection of atipamazole (Pfizer, New York, NY, USA; 0.55 mg). Evaluation of the female sibling was performed under general anesthesia by a board-certified veterinary ophthalmologist (EBB) and zoo veterinarian (MS) at the Metropolitan Veterinary Hospital, Akron, OH. Anesthesia was ‘chamber induced’ to effect and maintained with sevoflurane delivered in 100% oxygen via an endotracheal tube.

Ophthalmic diagnostics included Schirmer tear test I (Schirmer Tear Test, Schering-Plough Animal Health, Union, NJ, USA), rebound tonometry (TonoVet^®^; Icare, Espoo, Finland) and fluorescein stain (Flu-Glo^®^ Fluorescein sodium 1 mg stain; Akorn Inc., Buffalo Grove, IL, USA). Neuro-ophthalmic examinations included evaluation of direct and consensual pupillary light reflexes (PLRs) using a 3.5 V Finnoff transilluminator (Welch Allyn, Skaneateles Falls, NY, USA), assessment of dazzle reflex using white light directly from the fiberoptic cable of a slit lamp biomicroscope (SL-2; Kowa Optimed Inc, Torrance, CA, USA), behavioral testing of vision using menace response, and measurement of horizontal pupil diameter with Jameson caliper. Diffuse and focal illumination with a portable hand-held slit-lamp biomicroscope (SL-14; Kowa Col Ltd., Tokyo, Japan) was utilized to examine the anterior segment of both animals. Indirect ophthalmoscopy was performed with a wireless binocular indirect headset (12,500; Welch Allyn Inc., Skaneateles Falls, NY, USA) and a condensing lens (Pan Retinal 30D; Volk Optical Inc., Mentor, OH, USA). Mydriasis was not induced pharmacologically for examination of the fundus due to marked pupillary dilation present in both patients. The fundus of the male proband was photographed with a NIDEK NM-100 digital fundus camera (NIDEK CO. LTD., Aichi, Japan).

### Additional diagnostic procedures

A complete blood hematology, chemistry panel and urinalysis were completed in both affected African black-footed cats. Plasma taurine levels were also determined. Both affected cats completed electroretinography examinations (ERG) at their respective clinics. The ERG was completed in both cats under general anesthesia based on previous studies^60^. Both cats were dark adapted for at least 20 minutes prior to recording. Briefly, the ERG was performed utilizing a monopolar electrode-contact lens (ERG-jet; Nicolet Instruments, Madison, WI, USA) applied to the cornea with hypromellose 2.5% gel as a coupling agent (Gonak; Akorn, Inc., Buffalo Grove, IL, USA) and male subdermal platinum needle electrodes (FD-E2–24; Astro-Medical, Inc. Warwick, RI, USA). The ERG for the male proband was performed with a Handheld Multispecies ERG (HMsERG) (HMsERG Model 1000; RetVetCorp, Columbia, MO, USA) using the Quick Ret Check Protocol. The ERG for the female sibling was performed with a Retinographics unit (Retinographics Inc., Norwalk, CT). For both cats, mixed rod and cone responses were assessed with a standard flash intensity of 3 cd.s/m^2^. Mixed rod and cone responses of the male cat were also evaluated with a high intensity flash (10 cd.s/m^2^). Indirect blood pressure measurement was performed under general anesthesia in both cats.

All additional cats in the study with available DNA samples (n = 14), including the parents of the siblings, were reported to behave in a visually appropriate manner and failed to demonstrate any visual deficits.

### Genetic analyses

African black-footed cats (n = 16; eight males and eight female cats) from the captive populations at seven zoological parks were evaluated (Table 1). An aliquot of the DNA was submitted for commercial genotyping to the Veterinary Genetics Laboratory at the University of California, Davis, to screen for the three known variants associated with PRA in cats: *AIPL1, CRX* and *CEP290*. DNA for WGS was isolated by organic extraction from EDTA anti-coagulated whole blood collected from a trio of African black-footed cats, including the obligate carrier sire (205) and dam (208), and affected female offspring (247) (Table 1). The WGS libraries and sequencing were performed at the University of Missouri DNA Core on an Illumina HiSeq 2000 as previously described^11^. Cat WGS filtering and genome-wide variant calling were performed as previously described by Maverix Biomics, Inc. (San Mateo, CA)^11^ and as part of the 99 Lives whole genome sequencing initiative (http://felinegenetics.missouri.edu/99lives). Briefly, the WGS from the trio was aligned to the available annotated cat genome reference (Felis_catus 6.2 genome assembly) to identify variants^50^. All variants detected in the black-footed cat trio were filtered based on the known individuals’ phenotype and using the available variant dataset comprised of 52 whole genome sequenced cats, including one Pallas cat and 51 domestic cats. Variants were filtered as follows (1) considering variants only segregating in the black-footed cat trio, (2) excluding variants in common with black-footed cat and Pallas cat, (3) excluding variants shared in domestic and black-footed cats, (4) variants in concordant segregation with the autosomal recessive disease phenotype within the black-footed cat trio, and, (5) identifying variants in known retinal genes. A list of genes causing retinal diseases from the RetNet database (www.RetNet.org) was obtained and variants were visually investigated within those genes. Variants were prioritized by their effect on the protein using SNPeff (http://snpeff.sourceforge.net/SnpEff_manual.html#eff).

### IQ calmodulin-binding motif-containing protein-1 (IQCB1) variant genotyping

A direct sequencing assay was performed to screen a possible causative variant within the candidate gene *IQCB1* in all available black-footed cats with known phenotype (n = 16, Table 1). Two primers were designed on the variant flanking regions (IQCB1-FCCTCTCCTCATACACACTCAGAGTTA, IQCB1-R CGGTGTCTTTGGAGATAGTCATC) and PCR was performed at 58 °C annealing temperature. Cycles, reagents final concentrations, PCR purification and direct Sanger sequencing were performed as previously described^61^. Sanger sequencing was completed at the DNA Core at the University of Missouri on an ABI 3730 DNA Analyzer (Applied Biosystems, Foster City, CA).

### Mass spectroscopy variant genotyping

Candidate variants associated with a missense change in a RetNet gene were selected for genotyping. Assays were designed using the MassARRAY Assay Design included in the package Typer 4.0 (Agena Bioscience, San Diego, CA) according to manufacturer’s instructions. A multiplex consisting of seven SNPs in six different genes (*CHD3, CNGB1, DTHD1, RBP3, USH1C and CDH23*) was selected (Supplementary Table 1), genotyping was performed on all available samples using the Agena Biosciences iPLEX Gold Genotyping reagent set and products were typed with the MassARRAY System with *Nanodispenser RS1000* (Agena Bioscience).

### Assay performance and validation

All data was inspected with Typer Analyzer to assess assay quality and assays with weak or ambiguous signals were discarded. Next, the Autocluster algorithm of Typer Analyzer was selected and only assays with “Conservative” or “Moderate” calls were considered. Moreover, the three WGS black-footed cats functioned as positive controls for assay validation.

## Additional Information

**How to cite this article:** Oh, A. *et al*. Early-Onset Progressive Retinal Atrophy Associated with an *IQCB1* Variant in African Black-Footed Cats (*Felis nigripes*). *Sci. Rep.* **7**, 43918; doi: 10.1038/srep43918 (2017).

**Publisher's note:** Springer Nature remains neutral with regard to jurisdictional claims in published maps and institutional affiliations.

## Supplementary Material

Supplementary Information

## Figures and Tables

**Figure 1:**
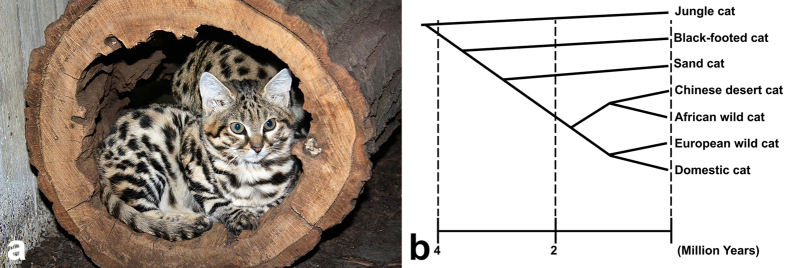
Phylogeny of the *Felis* lineage of small cats. (**a**) Image of a young black-footed cat (*Felis nigripes).* Courtesy of Cleveland Metroparks Zoo, Cleveland, OH. (**b**) This felid phylogeny suggests that the black-footed cat (*Felis nigripes)* had a common ancestor with the domestic cat (*Felis catus*) approximately 3 million years ago. Adapted from Li *et al*.^17^.

**Table 1:** Available DNA samples from the African black-footed cat captive population.

Animal ID	Gender	Date of Birth	Location	Phenotype	Genotype
205^a^	M	9/21/06	CMZ	Normal (parent of affected)	*IQCB1-*carrier
208^a^	F	2/20/07	CMZ	Normal (parent of affected)	*IQCB1-*carrier
222	F	3/12/09	KCZ	Normal	*wt*
223	F	3/12/09	OKL	Normal	*wt*
228	M	2/7/10	LOU	Normal (sibling of affected)	*IQCB1-*carrier
229	M	4/13/10	KCZ	Normal	*wt*
230	M	4/13/10	KCZ	Normal	*wt*
233	F	3/16/11	KCZ	Normal	*wt*
234	F	4/11/11	SDS	Normal	*wt*
235	M	4/17/11	KCZ	PRA-affected (proband)	*IQCB1-*mutant
236	M	4/17/11	OKL	Normal (sibling of affected)	*wt*
240	M	2/3/12	OMA	Normal	*IQCB1*-carrier
247^a^	F	4/2/12	CMZ	PRA-affected	*IQCB1-*mutant
248	F	4/2/12	CMZ	Normal (sibling of affected)	*IQCB1-*carrier
251	F	7/12/12	SCS	Normal	*wt*
258	M	8/8/14	OMA	Normal	*IQCB1-*carrier

Gender: M (male), F (female); location: Cleveland Metroparks Zoo (CMZ), Kansas City Zoo (KCZ), Louisville Zoological Garden (LOU), Oklahoma City Zoo (OKL), Omaha’s Henry Doorly Zoo (OMA), Riverbanks Zoo & Garden (SCS), San Diego Zoo Safari Park (SDS); genotype: *wild type (wt*), c.1282delCT variant is homozygous in *IQCB1-*mutant and heterozygous in *IQCB1-*carrier; ^a^whole genome sequenced cats. Animal identification is from the black-footed cat North American studbook managed by the species survival plan (B. Palmer, Denver Zoo, Denver, CO).

**Table 2:** WGS variants identified in 30x coverage of a black footed cat PRA trio.

Variant impact*	Analysis type**				
	Functional Class	WGS trio	Species- Specific	Phenotype Segregation	RetNet genes
High	Stop gain	678	454	8	—
	Start/Stop loss	134	330	—	—
	Splice donor/acceptor	2,547	88	22	—
	Exon deletion	4	1	—	—
	Frameshift	3,602	722	20	1
	Rare amino acid	—	—	—	—
Moderate	Codon alteration	1,462	711	13	—
	Missense	47,534	32,038	595	7
	Splice branch	—	—	—	—
	5′ or 3′ UTR Deletion	—	—	—	—
Low		89,718	59,119	859	13
Modifier		22,064,948	13,437,721	232,512	1,019

^*^Impact as defined by http://snpeff.sourceforge.net/SnpEff_manual.html#eff.

^**^Feline reference genome sequence (V6.2) is included in the analyses. Effect counts are higher than variant counts because they include the effects of each alternate allele on each nearby gene isoform.

**Table 3:** Comparison of genotype versus phenotype between seven moderate impact missense variants.

Animal ID	Gender	Phenotype	Genotype *IQCB1*	*CHD3*	*CNGB1*	*DTHD1_942*	*RBP3*	*USH1C*	*CDH23*	*DTHD1_144*
247^a^	F	PRA-affected	delCT/delCT	GG	TT	GG	AA	TT	TT	CC
205^a^	M	Normal	*wt/*delCT	AG	CT	GC	AG	CT	CT	CA
208^a^	F	Normal	*wt/*delCT	AG	CT	GC	AG	CT	CT	CA
235	M	PRA-affected	delCT/delCT	AA^b^	CC	GG	AG^b^	CT	TT	CC
240	M	Normal	*wt/*delCT	AA	CT	GG^b^	GG	CT	CT	CA
248	F	Normal	*wt/*delCT	/	CC	/	AG	TT	CC	/
258	M	Normal	*wt/*delCT	AA	TT^b^	GC	GG	CC	CC^b^	AA^b^
236	M	Normal	*wt/wt*	AA	CC	GC	AG	CT	CT^b^	CA^b^
230	M	Normal	*wt/wt*	AA	CC	CC	GG	CC	CC	AA
234	F	Normal	*wt/wt*	/	/	CC	GG	/	/	AA
251	F	Normal	*wt/wt*	AA	CC	CC	GG	CC	CT^b^	AA
229	M	Normal	*wt*	AA	CC	CC	GG	CT	CC	AA
222	F	Normal	*wt*	AA	CT	CC	GG	TT^b^	CT^c^	AA
233	F	Normal	*wt*	AA	CC	CC	GG	TT^b^	CC	CA^b^
223	F	Normal	*wt*	AA	CC	GC	GG	CT	CC	CA^c^

Gender: male (M), female (F); genotype: deleted 2 base pairs cytosine and thymine (delCT), *wild type (wt*), *IQCB1-*mutant (delCT/delCT), *IQCB1-*carrier (*wt/*delCT), *wild type (wt/wt*); nucleotides: guanine (G), adenine (A), thymine (T), cytosine (C); “/” no data due to sample error;

^a^whole genome sequenced trio of cats;

^b^missense variants excluded based on genotype versus phenotype mismatch;

^c^missense variants excluded when compared to the pedigree (Fig. 3). The moderate impact variant alleles are detected in unrelated black-footed cats that never interbred with the line of cats with vision loss.

## References

[b1] Miller N. A. . A 26-hour system of highly sensitive whole genome sequencing for emergency management of genetic diseases. Genome Med 7, 100, doi: 10.1186/s13073-015-0221-8 (2015). 26419432 PMC4588251

[b2] Saunders C. J. . Rapid whole-genome sequencing for genetic disease diagnosis in neonatal intensive care units. Science translational medicine 4, 135–154 (2012). 10.1126/scitranslmed.3004041 PMC4283791 23035047

[b3] Lupski J. R. . Whole-genome sequencing in a patient with Charcot-Marie-Tooth neuropathy. N. Engl. J. Med. 362, 1181–1191, doi: 10.1056/NEJMoa0908094 (2010). 20220177 PMC4036802

[b4] Michot P. . Whole-genome sequencing identifies a homozygous deletion encompassing exons 17 to 23 of the integrin beta 4 gene in a Charolais calf with junctional epidermolysis bullosa. Genet Sel Evol 47, 37, doi: 10.1186/s12711-015-0110-z (2015). 25935160 PMC4417276

[b5] Sayyab S. . Whole-Genome Sequencing of a Canine Family Trio Reveals a FAM83G Variant Associated with Hereditary Footpad Hyperkeratosis. G3 (Bethesda) 6, 521–527, doi: 10.1534/g3.115.025643 (2016). 26747202 PMC4777115

[b6] Bamshad M. J. . Exome sequencing as a tool for Mendelian disease gene discovery. Nat Rev Genet 12, 745–755, doi: 10.1038/nrg3031 (2011). 21946919

[b7] Rabbani B., Mahdieh N., Hosomichi K., Nakaoka H. & Inoue I. Next-generation sequencing: impact of exome sequencing in characterizing Mendelian disorders. J. Hum. Genet. 57, 621–632, doi: 10.1038/jhg.2012.91 (2012). 22832387

[b8] Daetwyler H. D. . Whole-genome sequencing of 234 bulls facilitates mapping of monogenic and complex traits in cattle. Nat. Genet. 46, 858–865, doi: 10.1038/ng.3034 (2014). 25017103

[b9] Viluma A., Sayyab S., Mikko S., Andersson G. & Bergstrom T. F. Evaluation of whole-genome sequencing of four Chinese crested dogs for variant detection using the ion proton system. Canine Genet Epidemiol 2, 16, doi: 10.1186/s40575-015-0029-2 (2015). 26457193 PMC4599337

[b10] Human Gene Mutation Database www.hgmd.cf.ac.uk Date of Access: 9.20.2016.

[b11] Gandolfi B. . COLQ variant associated with Devon Rex and Sphynx feline hereditary myopathy. Anim. Genet. 46, 711–715, doi: 10.1111/age.12350 (2015). 26374066 PMC4637250

[b12] Lyons L. A. . Whole genome sequencing in cats, identifies new models for blindness in AIPL1 and somite segmentation in HES7. BMC Genomics 17, 265, doi: 10.1186/s12864-016-2595-4 (2016). 27030474 PMC4815086

[b13] Cho Y. S. . The tiger genome and comparative analysis with lion and snow leopard genomes. Nat Commun 4, 2433, doi: 10.1038/ncomms3433 (2013). 24045858 PMC3778509

[b14] Xu X. . The genetic basis of white tigers. Curr. Biol. 23, 1031–1035, doi: 10.1016/j.cub.2013.04.054 (2013). 23707431

[b15] Silwa A. Home range size and social organisation of black-footed cats (Felis nigripes). Mamm biol 69, 96–107 (2004).

[b16] Johnson W. E. . The late Miocene radiation of modern Felidae: a genetic assessment. Science 311, 73–77, doi: 10.1126/science.1122277 (2006). 16400146

[b17] Li G., Davis B., Eizirik E. & Murphy W. Phylogenomic evidence for ancient hybridization in the genomes of living cats (Felidae). Genome Res., doi: 10.1101/gr.186668.114 (2015). PMC4691742 26518481

[b18] Mattern M. Y. & McLennan D. A. Phylogeny and speciation of felids. Cladistics 16, 232–253, doi: 10.1111/J.1096-0031.2000.Tb00354.X (2000). 34902955

[b19] CITES (Convention on International Trade in Endangered Species of Wild Fauna and Flora) https://cites.org/eng Date of Access: 9.29.2016.

[b20] Nowell K., Jackson P. & IUCN/SSC Cat Specialist Group. Wild cats: status survey and conservation action plan(IUCN, 1996).

[b21] U. S. Fish and Wildlife Service https://www.fws.gov/ Date of Access: 9.29.2016.

[b22] Olbricht G. & Sliwa A. *In situ* and *ex situ* observations and management of black-footed cats Felis nigripes. Intl Zoo Yearbook 35, 81–89 (1997).

[b23] Terio K. A., O’Brien T., Lamberski N., Famula T. R. & Munson L. Amyloidosis in black-footed cats (Felis nigripes). Vet. Pathol. 45, 393–400, doi: 10.1354/vp.45-3-393 (2008). 18487501

[b24] Petersen-Jones S. M. A review of research to elucidate the causes of the generalized progressive retinal atrophies. Vet. J. 155, 5–18 (1998). 9455155 10.1016/s1090-0233(98)80028-2

[b25] Ofri R. . Characterization of an Early-Onset, Autosomal Recessive, Progressive Retinal Degeneration in Bengal Cats. Invest Ophthalmol Vis Sci 56, 5299–5308, doi: 10.1167/iovs.15-16585 (2015). 26258614 PMC4539567

[b26] Menotti-Raymond M. . Mutation in CEP290 discovered for cat model of human retinal degeneration. The Journal of heredity 98, 211–220, doi: 10.1093/jhered/esm019 (2007). 17507457

[b27] Menotti-Raymond M. . Mutation discovered in a feline model of human congenital retinal blinding disease. Invest. Ophthalmol. Vis. Sci. 51, 2852–2859, doi: 10.1167/iovs.09-4261 (2010). 20053974 PMC2891453

[b28] Alhaddad H. . Genome-wide association and linkage analyses localize a progressive retinal atrophy locus in Persian cats. Mamm Genome 25, 354–362, doi: 10.1007/s00335-014-9517-z (2014). 24777202 PMC4105591

[b29] Barnett K. C. & Curtis R. Autosomal dominant progressive retinal atrophy in Abyssinian cats. J. Hered. 76, 168–170 (1985). 3998438 10.1093/oxfordjournals.jhered.a110058

[b30] Curtis R., Barnett K. C. & Leon A. An early-onset retinal dystrophy with dominant inheritance in the Abyssinian cat. Clinical and pathological findings. Invest. Ophthalmol. Vis. Sci. 28, 131–139 (1987). 3804643

[b31] Leon A. & Curtis R. Autosomal dominant rod-cone dysplasia in the Rdy cat. 1. Light and electron microscopic findings. Exp. Eye Res. 51, 361–381 (1990). 2209749 10.1016/0014-4835(90)90149-o

[b32] Leon A., Hussain A. A. & Curtis R. Autosomal dominant rod-cone dysplasia in the Rdy cat. 2. Electrophysiological findings. Exp. Eye Res. 53, 489–502 (1991). 1936184 10.1016/0014-4835(91)90166-c

[b33] Chong N. H., Alexander R. A., Barnett K. C., Bird A. C. & Luthert P. J. An immunohistochemical study of an autosomal dominant feline rod/cone dysplasia (Rdy cats). Exp. Eye Res. 68, 51–57, doi: 10.1006/exer.1998.0580 (1999). 9986741

[b34] Narfstrom K. Hereditary progressive retinal atrophy in the Abyssinian cat. J. Hered. 74, 273–276 (1983). 6886375 10.1093/oxfordjournals.jhered.a109782

[b35] Narfstrom K. Progressive retinal atrophy in the Abyssinian cat. Clinical characteristics. Invest. Ophthalmol. Vis. Sci. 26, 193–200 (1985). 3972501

[b36] Narfstrom K. & Nilsson S. E. Progressive retinal atrophy in the Abyssinian cat. Electron microscopy. Invest. Ophthalmol. Vis. Sci. 27, 1569–1576 (1986). 3771137

[b37] Narfstrom K., Arden G. B. & Nilsson S. E. Retinal sensitivity in hereditary retinal degeneration in Abyssinian cats: electrophysiological similarities between man and cat. Br. J. Ophthalmol. 73, 516–521 (1989). 2757991 10.1136/bjo.73.7.516 PMC1041792

[b38] Menotti-Raymond M. . Widespread retinal degenerative disease mutation (rdAc) discovered among a large number of popular cat breeds. Vet. J. 186, 32–38, doi: 10.1016/j.tvjl.2009.08.010 (2010). 19747862 PMC6984347

[b39] Rah H., Maggs D. J., Blankenship T. N., Narfstrom K. & Lyons L. A. Early-onset, autosomal recessive, progressive retinal atrophy in Persian cats. Invest Ophthalmol Vis Sci 46, 1742–1747, doi: 10.1167/iovs.04-1019 (2005). 15851577

[b40] Rah H., Maggs D. J. & Lyons L. A. Lack of genetic association among coat colors, progressive retinal atrophy and polycystic kidney disease in Persian cats. J Feline Med Surg 8, 357–360, doi: 10.1016/j.jfms.2006.04.002 (2006). 16777456 PMC10822235

[b41] Narfstrom K., Holland Deckman K. & Menotti-Raymond M. The domestic cat as a large animal model for characterization of disease and therapeutic intervention in hereditary retinal blindness. Journal of ophthalmology 2011, 906943, doi: 10.1155/2011/906943 (2011). 21584261 PMC3090773

[b42] Cullen C. L., Lim C. & Sykes J. Tear film breakup times in young healthy cats before and after anesthesia. Vet Ophthalmol 8, 159–165, doi: 10.1111/j.1463-5224.2005.00347.x (2005). 15910368

[b43] Margadant D. L., Kirkby K., Andrew S. E. & Gelatt K. N. Effect of topical tropicamide on tear production as measured by Schirmer’s tear test in normal dogs and cats. Vet Ophthalmol 6, 315–320 (2003). 14641829 10.1111/j.1463-5224.2003.00313.x

[b44] Arnett B. D., Brightman A. H. 2nd & Musselman E. E. Effect of atropine sulfate on tear production in the cat when used with ketamine hydrochloride and acetylpromazine maleate. J Am Vet Med Assoc 185, 214–215 (1984). 6746393

[b45] Ghaffari M. S., Malmasi A. & Bokaie S. Effect of acepromazine or xylazine on tear production as measured by Schirmer tear test in normal cats. Vet Ophthalmol 13, 1–3, doi: 10.1111/j.1463-5224.2009.00738.x (2010). 20149168

[b46] Miller P. E., Pickett J. P., Majors L. J. & Kurzman I. D. Evaluation of two applanation tonometers in cats. Am. J. Vet. Res. 52, 1917–1921 (1991). 1785739

[b47] Crispin S. M. & Mould J. R. Systemic hypertensive disease and the feline fundus. Vet Ophthalmol 4, 131–140 (2001). 11422995 10.1046/j.1463-5224.2001.00190.x

[b48] Jepson R. E. Feline systemic hypertension: Classification and pathogenesis. Journal of feline medicine and surgery 13, 25–34, doi: 10.1016/j.jfms.2010.11.007 (2011). 21215946 PMC10845409

[b49] Stepien R. L. Feline systemic hypertension: Diagnosis and management. Journal of feline medicine and surgery 13, 35–43, doi: 10.1016/j.jfms.2010.11.008 (2011). 21215947 PMC10845410

[b50] Montague M. J. . Comparative analysis of the domestic cat genome reveals genetic signatures underlying feline biology and domestication. Proceedings of the National Academy of Sciences of the United States of America 111, 17230–17235, doi: 10.1073/pnas.1410083111 (2014). 25385592 PMC4260561

[b51] Otto E. A. . Nephrocystin-5, a ciliary IQ domain protein, is mutated in Senior-Loken syndrome and interacts with RPGR and calmodulin. Nat. Genet. 37, 282–288, doi: 10.1038/ng1520 (2005). 15723066

[b52] Hildebrandt F. & Zhou W. Nephronophthisis-associated ciliopathies. J. Am. Soc. Nephrol. 18, 1855–1871, doi: 10.1681/asn.2006121344 (2007). 17513324

[b53] Estrada-Cuzcano A., Roepman R., Cremers F. P., den Hollander A. I. & Mans D. A. Non-syndromic retinal ciliopathies: translating gene discovery into therapy. Hum. Mol. Genet. 21, R111–124, doi: 10.1093/hmg/dds298 (2012). 22843501

[b54] Simms R. J., Hynes A. M., Eley L. & Sayer J. A. Nephronophthisis: a genetically diverse ciliopathy. International journal of nephrology 2011, 527137, doi: 10.4061/2011/527137 (2011). 21660307 PMC3108105

[b55] Schafer T. . Genetic and physical interaction between the NPHP5 and NPHP6 gene products. Hum. Mol. Genet. 17, 3655–3662, doi: 10.1093/hmg/ddn260 (2008). 18723859 PMC2802281

[b56] Hildebrandt F., Attanasio M. & Otto E. Nephronophthisis: disease mechanisms of a ciliopathy. J. Am. Soc. Nephrol. 20, 23–35, doi: 10.1681/asn.2008050456 (2009). 19118152 PMC2807379

[b57] Goldstein O. . IQCB1 and PDE6B mutations cause similar early onset retinal degenerations in two closely related terrier dog breeds. Invest. Ophthalmol. Vis. Sci. 54, 7005–7019, doi: 10.1167/iovs.13-12915 (2013). 24045995 PMC3809947

[b58] Downs L. M. . Overlap of abnormal photoreceptor development and progressive degeneration in Leber congenital amaurosis caused by NPHP5 mutation. Hum. Mol. Genet., doi: 10.1093/hmg/ddw254 (2016). PMC5291197 27506978

[b59] Wiik A. C. . A deletion in nephronophthisis 4 (NPHP4) is associated with recessive cone-rod dystrophy in standard wire-haired dachshund. Genome Res. 18, 1415–1421, doi: 10.1101/gr.074302.107 (2008). 18687878 PMC2527698

[b60] Labelle A. L., Hamor R. E., Narfstrom K. & Breaux C. B. Electroretinography in the western gray kangaroo (Macropus fuliginosus). Vet Ophthalmol 13 Suppl, 41–46, doi: 10.1111/j.1463-5224.2010.00810.x (2010). 20840089

[b61] Gandolfi B. . First WNK4-hypokalemia animal model identified by genome-wide association in Burmese cats. PloS one 7, e53173, doi: 10.1371/journal.pone.0053173 (2012). 23285264 PMC3532348

