# Corrigendum: Early-Onset Progressive Retinal Atrophy Associated with an *IQCB1* Variant in African Black-Footed Cats (*Felis nigripes*)

**DOI:** 10.1038/srep46978

**Published:** 2018-05-18

**Authors:** Annie Oh, Jacqueline W. Pearce, Barbara Gandolfi, Erica K. Creighton, William K. Suedmeyer, Michael Selig, Ann P. Bosiack, Leilani J. Castaner, Rebecca E. H. Whiting, Ellen B. Belknap, Leslie A. Lyons, 99 Lives Consortium, Danielle Aderdein, Paulo C. Alves, Gregory S. Barsh, Holly C. Beale, Adam R. Boyko, Marta G. Castelhano, Patricia Chan, N. Matthew Ellinwood, Dorian J. Garrick, Christopher R. Helps, Christopher B. Kaelin, Tosso Leeb, Hannes Lohi, Maria Longeri, Richard Malik, Michael J. Montague, John S. Munday, William J. Murphy, Niels C. Pedersen, Max F. Rothschild, William F. Swanson, Karen A. Terio, Rory J. Todhunter, Wesley C. Warren

Scientific Reports 7: Article number: 4391810.1038/srep43918; published online: 03 21 2017; updated: 05 18 2018

The Acknowledgements section in this Article is incomplete.

“Funding was provided by The University of Missouri, College of Veterinary Medicine Clinician Scientist Grant 2014 (JWP). Funding for the 99 Lives Cat Genome Sequencing Initiative has been provided by the Winn Feline Foundation and the George Sydney and Phyllis Redman Miller Trust (MT–13–010), the National Geographic Society Education Foundation (2P-14), the University of Missouri Gilbreath-McLorn Endowment (LAL), contributions from the 99 Lives Consortium participants, donations from Associazione Nazionale Felina Italiana (ANFI), Zoetis, Orivet Genetic Pet Care, Langford Veterinary Services, the World Cat Federation, and public donations. We appreciate the provision of cat DNA samples by Cristy Bird, Bruno Chomel, Kyung Sik Kim, Nassem N. Naimi of Best Friend Veterinary Clinic in Amman, Jordan, Julie Pomerantz, John Snape, Susanne and Claus Wehnert, Nancy Carpenter at Utah’s Hogle Zoo, Ashleigh Lutz-Nelson at San Francisco Zoo & Gardens and Julie Feinstein at the American Museum of Natural History. The authors would like to acknowledge Ms. Barbara Palmer for her assistance with studbook data, and Ms. Karen Clifford for pedigree graphics and construction. We appreciate the assistance of the research team at Maverix Biomics, Inc., especially Michael Fitzsimmons.”

should read:

“Funding was provided by The University of Missouri, College of Veterinary Medicine Clinician Scientist Grant 2014 (JWP). Funding for the 99 Lives Cat Genome Sequencing Initiative has been provided by the Winn Feline Foundation and the George Sydney and Phyllis Redman Miller Trust (MT–13–010), the National Geographic Society Education Foundation (2P-14), the University of Missouri Gilbreath-McLorn Endowment (LAL), the Cornell Feline Health Center (ARB, MGC, RJT), contributions from the 99 Lives Consortium participants, donations from Associazione Nazionale Felina Italiana (ANFI), Zoetis, Orivet Genetic Pet Care, Langford Veterinary Services, the World Cat Federation, and public donations. We appreciate the provision of cat DNA samples by Cristy Bird, Bruno Chomel, Kyung Sik Kim, Nassem N. Naimi of Best Friend Veterinary Clinic in Amman, Jordan, Julie Pomerantz, John Snape, Susanne and Claus Wehnert, Nancy Carpenter at Utah’s Hogle Zoo, Ashleigh Lutz-Nelson at San Francisco Zoo & Gardens and Julie Feinstein at the American Museum of Natural History. The authors would like to acknowledge Ms. Barbara Palmer for her assistance with studbook data, and Ms. Karen Clifford for pedigree graphics and construction. We appreciate the assistance of the research team at Maverix Biomics, Inc., especially Michael Fitzsimmons.”

