# Supplementary material for: Early-Onset Progressive Retinal Atrophy Associated with an IQCB1 Variant in African Black-Footed Cats (Felis nigripes)
Source: Sci Rep. 2017 Mar 21;7:43918. doi: 10.1038/srep43918 (PMC5359545; doi:10.1038/srep43918)
Supplement: Supplementary Information [file srep43918-s1.pdf]

Early-Onset Progressive Retinal Atrophy Associated with an *IQCB1* Variant in African Black-Footed Cats (*Felis nigripes)*

Running title: Progressive Retinal Atrophy and *IQCB1*

Annie Oh[1,†], Jacqueline W. Pearce[1*], Barbara Gandolfi[1,†], Erica K. Creighton[1], William K. Suedmeyer[2¶], Michael Selig[3¶], Ann P. Bosiack[4], Leilani J. Castaner[1], Rebecca E.H. Whiting[1], Ellen B. Belknap[5], Leslie A. Lyons[1], 99 Lives Consortium[6-27]

[1]Department of Veterinary Medicine and Surgery, College of Veterinary Medicine, University of Missouri, Columbia, Missouri, United States of America

[2]Kansas City Zoo, Kansas City, Missouri, United States of America

[3]Cleveland Metroparks Zoo, Cleveland, Ohio, United States of America

[4]Animal Eye Care of Richmond LLC, Midlothian, Virginia, United States of America

[5]Metropolitan Veterinary Referral Hospital, Akron, Ohio, United States of America

*Corresponding author:

E-mail: pearcej@missouri.edu (JWP)

[†]These authors contributed equally to this work.

Supplementary Table 1. List of 50 genes containing high impact variants genome-wide.

Gene	Name	Chromosome	Retinal degeneration
CALCR	Calcitonin receptor	A2	N
CASS4	Cas scaffolding protein family member 4	A3	N
CCDC114	Coiled-coil domain containing 114	E2	Y
DOCK5	Dedicator of cytokinesis 5	B1	N
ENSFCAG00000005293 (UBTFL1)	Upstream binding transcription factor, RNA polymerase I-like 1	D1	N
ENSFCAG00000011443	Uncharacterized protein	A2	-
ENSFCAG00000021906	Pseudogene	D4	-
ENSFCAG00000022528	Pseudogene	A1	-
ENSFCAG00000022562	Olfactory receptor	D1	N
ENSFCAG00000023179 (EIF4G1)	Eukaryotic translation initiation factor 4 gamma 1	B4	N
ENSFCAG00000023724	Uncharacterized protein	C1	-
ENSFCAG00000023754	Uncharacterized protein	B4	-
ENSFCAG00000025216	Olfactory receptor	D1	N
ENSFCAG00000026054	Uncharacterized protein	B3	-
ENSFCAG00000027461	Uncharacterized protein	F1	-
ENSFCAG00000027565	Uncharacterized protein	D3	-
ENSFCAG00000027642	Olfactory receptor	D1	N
ENSFCAG00000027859	Uncharacterized protein	D1	-
ENSFCAG00000028046	Uncharacterized protein	B3	-
ENSFCAG00000029147	Uncharacterized protein	X	-
ENSFCAG00000029400	Uncharacterized protein	A3	-
ENSFCAG0000002994	Uncharacterized protein	E2	-

3			
ENSFCAG00000030492	Uncharacterized protein	F2	-
ENSFCAG00000031149	Uncharacterized protein	X	-
IGSF9B	Immunoglobulin superfamily, member 9B	D1	N
IQCB1	**IQ motif containing B1**	**C2**	**Y**
ITGB6	Integrin, beta 6	C1	N
KIAA2026	Uncharacterized protein	D4	-
KMT2D	Lysine (K)-specific methyltransferase 2D	B4	N
LAMC2	Laminin, gamma 2	F1	N
LFNG	LFNG O-fucosylpeptide 3-beta-N-acetylglucosaminyltransferase	E3	N
LIMK1	LIM domain kinase 1	E3	N
NCKAP1L	NCK-associated protein 1-like	B4	N
NLE1	Notchless homolog 1	E1	N
NOL3	Nucleolar protein 3 (apoptosis repressor with CARD domain)	E2	N
PABPC4	Polyadenylate-binding protein	C1 or F2	N
PARD3	Par-3 family cell polarity regulator	B3	N
PLA2G10	Phospholipase A2, group X	E3	N
PODNL1	Podocan-like 1	A2	N
PSPN	Persephin	A2	N
PTPRU	Protein tyrosine phosphatase, receptor type, U	C1	N
RASGRF1	Ras protein-specific guanine nucleotide-releasing factor 1	B3	Y
RFC2	Replication factor C	E3	N
RRAD	Ras-related associated with diabetes	E2	N
SERHL2	Serine hydrolase-like 2	B4	N
SLC43A3	Solute carrier family 43, member 3	D1	N
SPTBN4	Spectrin, beta, non-erythrocytic 4	E2	N
TRPM4	Transient receptor potential cation channel, subfamily M, member 4	E2	N
WDR66	WD repeat domain 66	D3	N
ZP1	Zona pellucida glycoprotein 1	D1	N

Retinal degeneration: yes (Y), no (N), - (Unknown).

Supplementary Table 2. MassARRAY genotyping primer sequences.

**Primer name**	**5' -> 3' sequence**
CDH23-forward	ACGTTGGATGTGACGATGAAGGAGAAGACG
CDH23-reverse	ACGTTGGATGAAGAACTTCCATCTGCAGCC
CDH23-extension	CTTCCATCTGCAGCCTGACGGG
CDH3-forward	ACGTTGGATGGGTGAACATGAGGTCATGTG
CDH3-reverse	ACGTTGGATGCCATCAACACCTACAATGGG
CDH3-extension	GTGGGTGGTTGCTTACTCT
CNGB1-forward	ACGTTGGATGAAGATGCCGATGCTGACGAC
CNGB1-reverse	ACGTTGGATGATTTGGCAGGAGAAGGTGGC
CNGB1-extension	GAGGGTTCTCCAAGATGA
DTHD1_144-forward	ACGTTGGATGGTCTTCTTTTTCCACTAAGTC
DTHD1_144-reverse	ACGTTGGATGGTCATTATACGAACCAGGTC
DTHD1_144-extension	CCAGGTCCAGTTAAAAAGAAA
DTHD1_942-forward	ACGTTGGATGATCGGTGGAATTTGGGAGTG
DTHD1_942-reverse	ACGTTGGATGCATCAAACTGAAGACCCCTG
DTHD1_942-extension	GGTGACCTCACAGAACA
RBP3-forward	ACGTTGGATGAGGATGAGCCTCCGGAGGTA
RBP3-reverse	ACGTTGGATGCCTGAAACATAGAGACCACC
RBP3-extension	CCTCAGCTCCGGGCACC
USH1C-forward	ACGTTGGATGTGTAGCTCTTCTCTTCCCTC
USH1C-reverse	ACGTTGGATGAGGAGGAACCTGTGTCTATG
USH1C-extension	CGGCTGGGGGCTTTCGG
